# Impact of Pneumococcal Vaccination on Nasopharyngeal Carriage of *Streptococcus pneumoniae* and Microbiota Profiles in Preschool Children in South East Poland

**DOI:** 10.3390/vaccines10050791

**Published:** 2022-05-17

**Authors:** Karolina Kielbik, Aleksandra Pietras, Joanna Jablonska, Adrian Bakiera, Anna Borek, Grazyna Niedzielska, Michal Grzegorczyk, Ewelina Grywalska, Izabela Korona-Glowniak

**Affiliations:** 1 Department of Pharmaceutical Microbiology, Medical University of Lublin, 20-093 Lublin, Poland; abakiera98@gmail.com (A.B.); aniaborek97@wp.pl (A.B.); 2 Department of Pediatric Otolaryngology, Phoniatrics and Audiology, Medical University of Lublin, 20-093 Lublin, Poland; marciniak.alex@gmail.com (A.P.); joanna.janowska@gmail.com (J.J.); grazyna.niedzielska@wp.pl (G.N.); 3 Department of Rehabilitation and Physiotherapy, Medical University of Lublin, 20-081 Lublin, Poland; michal.grzegorczyk@umlub.pl; 4 Department of Experimental Immunology, Medical University of Lublin, 20-093 Lublin, Poland; ewelina.grywalska@umlub.pl

**Keywords:** *Streptococcus pneumoniae*, pneumococcal conjugate vaccine, serotypes, nasopharyngeal microbiota, antibiotic resistance patterns

## Abstract

In 2017, Poland introduced the 10-valent pneumococcal conjugate vaccine (PCV) into its national immunization schedule. This prospective study was conducted between March and June 2020 to determine the impact of vaccination on prevalence of the nasopharyngeal carriage of *S. pneumoniae* in 176 healthy children and to determine how conjugate vaccines indirectly affect colonization of nasopharyngeal microbiota. Pneumococcal isolates were analyzed by serotyping and antimicrobial resistance tests. Nasopharyngeal microbiota were detected and identified using the culture method and real-time PCR amplification primers and hydrolysis-probe detection with the 16S rRNA gene as the target. In the vaccinated group of children, colonization was in 24.2% of children, compared to 21.4% in the unvaccinated group. Serotypes 23A and 23B constituted 41.5% of the isolates. Serotypes belonging to PCV10 and PCV13 constituted 4.9% and 17.1% of the isolates, respectively. *S. pneumoniae* isolates were resistant to penicillin (34.1%), erythromycin (31.7%), and co-trimoxazole (26.8%). Microbial DNA qPCR array correlated to increased amounts of *Streptococcus mitis* and *S. sanguinis* in vaccinated children, with reduced amounts of *C. pseudodiphtericum*, *S. aureus*, and *M. catarrhalis*. Introduction of PCV for routine infant immunization was associated with significant reductions in nasopharyngeal carriage of PCV serotypes and resistant strains amongst vaccine serotypes, yet carriage of non-PCV serotypes increased modestly, particularly serotype 23B.

## 1. Introduction

*Streptococcus pneumoniae* is one of the major bacterial pathogens colonizing the nasopharynx, mainly asymptomatically; colonization usually precedes infection. Nasopharyngeal carriage in healthy children is a major factor in the horizontal transmission of pneumococcal strains, especially in children attending daycare centers (DCCs), or to other family members [1]. Moreover, pneumococcal nasopharyngeal isolates reflect the strains currently circulating in the community. Pneumococcus is also an important human pathogen responsible for severe infections: meningitis, bacteremia, and pneumonia with bacteremia classified as an invasive pneumococcal disease (IPD) as well as relatively mild upper respiratory tract infections, such as acute otitis media (AOM), both in children and adults [2]. This duality of commensalism and pathogenicity is why *S. pneumoniae* is alternatively defined as a pathobiont [3]; 100 pneumococcal serotypes based on the capsular polysaccharide have been identified and have spread worldwide. These serotypes vary in terms of incidence, disease manifestation, antibiotic resistance, and activate the host immune system. Several of them, so-called ‘pediatric serotypes’, 6A, 6B, 9V, 14, 15A, 19F, 19A, 23F, were most often detected in serious infections in children [4,5,6].

Previous studies have highlighted a number of risk factors for *S. pneumoniae* carriage in children, including DCC attendance, exposure in the family, number of siblings, passive smoking, age, and the female gender [5,7]. Current pneumococcal conjugate vaccines (PCVs) only target a limited number of serotypes, especially those commonly causing IPD in young children. The WHO recommends that all countries introduce PCVs, which interrupt the transmission of antibiotic-resistant pneumococci and, thus, decrease the burden of disease caused by antibiotic-resistant isolates in immunized children [8]. Therefore, studies on the impacts of these vaccines on antibiotic resistance and serotype distribution should focus on pneumococci from nasopharyngeal carriage [9]. Before PCVs were introduced in 2000, the global annual number of serious pneumococcal disease cases in children under five years of age was around 14.5 million [10]. When PCVs were implemented in 129 countries, this number decreased to 9.2 million in the 2015 cases, caused by vaccine serotypes in vaccinated children and other age groups due to herd immunity [8,11]. However, shifts in colonization and disease toward non-vaccine serotypes and other potential pathogens have been described [12,13,14]. Due to the dynamically changing epidemiological status across the world, epidemiological data, such as serotype variability, serotype replacement, and antibiotic resistance of pneumococci should be monitored. Recent studies indicate that the carriage rates of other co-colonizing pathogens, such as *Staphylococcus aureus* and nontypeable *Hemophilus influenzae*, may also be changed after PCV introduction [15,16].

In 2017, Poland introduced the 10-valent PCV (PCV-10) into its national immunization schedule. For approximately a decade prior to this introduction, PCV was recommended to all children (for a fee) and was given free-of-charge to high-risk groups. The 13-valent pneumococcal conjugate vaccine (PCV-13) was made available for the private market and often used [17]. The basic vaccination schedule consists of three or four doses, according to the country’s recommendation. In Poland, it consists of two primary doses followed by a supplementary dose of the PCV-10, with some modifications in case of specific risk factors.

Poland was one of the last countries in Europe to introduce free-of-charge, mandatory vaccinations against pneumococcal disease. The introduction of routine childhood immunization schedules of PCV10 or PCV13 imposes the need for monitoring the utility of PCV vaccines as an effective prevention of pneumococcal colonization and infections. The aim of the study was to determine the prevalence of nasopharyngeal carriage of *S. pneumoniae* in children aged 1–6 years who were vaccinated with PCVs in comparison to non-vaccinated children and how conjugate vaccines indirectly affect colonization of nasopharyngeal microbiota.

## 2. Materials and Methods

### 2.1. Patients

This prospective, multi-center, cross-sectional observational study was conducted over the winter and spring seasons between March and June 2020, involving 176 children between 1 and 6 years old (mean 4.1 ± SD 1.4) who belonged to different social groups in Lublin Voivodship. They were selected from two location types: pediatric ambulatory care clinics (n = 104) and kindergartens (n = 72). Considering that the size of the children’s population (between 1 and 6 years old) in the tested region is approximately 200,000, the margin of error of the measured value at the 95% confidence interval was ±7.38% when testing 176 children. The protocol was reviewed and approved by the Bioethics Committee of the Medical University of Lublin (KE-0254/45/2020) and performed in compliance with the Helsinki declaration. Written informed consent was received from each patient.

### 2.2. Laboratory Procedures

A nasopharyngeal sample using a sterile swab with Amies medium (Swabs^®^, DELTALAB, Barcelona, Spain) was obtained from each child who participated in the study. Swabs were inoculated on the selective Mueller-Hinton agar with 5% sheep blood and 5 mg/L of gentamicin for selective cultivation of streptococci. The streaked agar plates were incubated aerobically at 35 °C in 5% CO_2_ enriched atmosphere for 24 to 48 h. Pneumococci were identified by colony morphology, susceptibility to optochin (5 μg), and bile solubility; identification was confirmed by a slide agglutination test DrySpot™ Pneumo Test (Oxoid Ltd., Hampshire, UK). All isolates were serotyped by means of the Quellung reaction using antisera provided by Statens Serum Institute (Copenhagen, Denmark) and confirmed by a sequential multiplex PCR according to CDC recommendations [18]. The isolates were confirmed by the restriction digest (BsaAI) of the PCR product of a lytA gene encoding the autolysin enzyme specific to *S. pneumoniae* [19]. Susceptibility of the isolates to oxacillin, erythromycin, tetracycline, clindamycin, norfloxacin (isolates categorized as screen negative can be reported susceptible to moxifloxacin and as “susceptible increased exposure” (I) to levofloxacin), vancomycin, and trimethoprim–sulfamethoxazole was determined by the disk diffusion method of Bauer and Kirby on Mueller-Hinton agar with 5% mechanically-defibrinated horse blood and 20 mg/L β-NAD. Results were interpreted according to the European Committee on Antimicrobial Susceptibility Testing recommendations (EUCAST, 2020). Isolates exhibiting a zone of >20 mm around a 1 μg oxacillin disk were reported as penicillin susceptible *S. pneumoniae* (PSSP); isolates exhibiting a zone of ≤20 mm were further tested by the E-test (AB Biodisk, Sweden), following the manufacturer’s instructions, to determine minimal inhibitory concentration (MIC) for benzylpenicillin. Isolates with MIC ≤ 0.064 mg/L were considered as fully susceptible to benzylpenicillin; isolates with MIC > 0.064 mg/L were called penicillin non-susceptible *S. pneumoniae* (PNSSP). Multidrug-resistant isolates of *S. pneumoniae* (MDR-SP) were defined as having resistance to at least three different classes of antibiotics. *S. pneumoniae* ATCC 49619 was used as a control strain in the antimicrobial susceptibility tests.

### 2.3. Real-Time PCR Analysis

The nasopharyngeal samples were stored at −70 °C until RT-PCR could be performed. DNA from samples were extracted using Genomic DNA purification with QIAamp DNA Mini Kit (Qiagen, Germantown, MA, USA) according to the manufacturer’s instructions and analyzed with the Custom Microbial DNA qPCR Array (Qiagen, Germantown, MA, USA). Real-Time PCR assays were performed (Light Cycler 96, Roche, Basel, Switzerland) using the 16S rRNA gene as the target, and we used PCR amplification primers and hydrolysis-probe detection, which increased the specificity of each assay. Each Microbial DNA qPCR Array plate analyzed one sample for 21 species (NCBI Tax ID)/gene at a time. Pan-bacteria assays that detect a broad range of bacterial species were included to serve as positive controls for the presence of bacterial DNA. Relative profiling applications were measured for host genomic DNA and overall bacterial load. Inclusion of these analyses allows the user to normalize sample input using ΔΔCT.

### 2.4. Statistical Analysis

The statistical analysis was performed with Tibco Statistica 13.3 (StatSoft, Palo Alto, CA, USA). The values of the parameters are presented as median, minimum, and maximum values. Normal distributions of continuous variables were tested using the Shapiro–Wilk test. The Mann–Whitney U-test was used for independent variable comparisons. Kruskal–Wallis ANOVA and multiple comparisons of mean rank (as post-hoc analysis) were applied to analyze differences between more than two groups. The power and direction of association between pairs of continuous variables (studied groups) were determined using Spearman’s coefficient of rank correlation. The distribution of discrete variables in groups were compared with Pearson’s chi-square test or the Fisher’s exact test. The multivariate data analyses were carried out using the SIMCA 16 (v16.0.2, Umetrics, Sweden). Relative bacterial species abundance in the nasopharynx were calculated according to the real-time PCR data analyzing protocol [20]. Principal component analysis (PCA) was used to identify similarities and differences between analyzed samples. Data were scaled to unit variance and centered. The hierarchical cluster analysis (HCA) and partial last square discriminant analysis model (PLS-DA) were used for root sample classification and predictions. 

## 3. Results

A total of 176 children were enrolled in the study, 97 boys and 79 girls. Among them, 25 children aged 1–2 years old, 75 aged 3–4 years, and 76 aged 5–6 years. Demographic data of the studied children are shown in Table 1.

### 3.1. Serotype Distribution and Vaccine Coverage

A total of 41 isolates were recovered from the nasopharynx of 176 healthy children aged 1 to 6. Pneumococcal colonization was observed in 23.3% children. In the vaccinated group, colonization was in 24.2% children, compared to 21.4% in the unvaccinated group. Carriage increased from 13.2% among 5–6-year-old children to 33.3% in 3–4-year-old children (*p* = 0.0038). 

**Figure 1:**
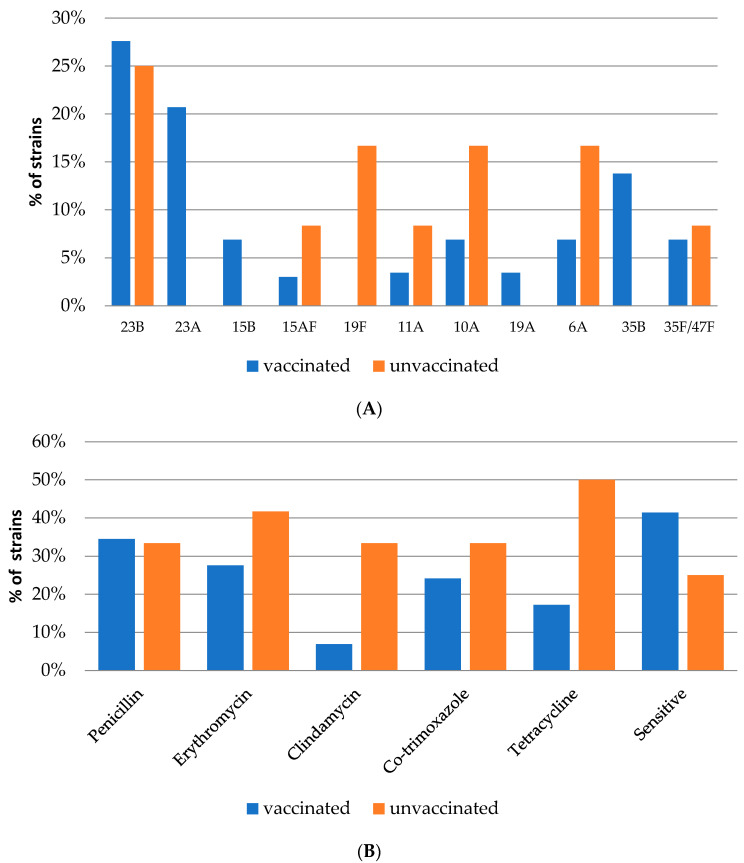
Serotype-specific prevalence (**A**) and antibiotic resistance (**B**) among pneumococci carriage isolates in unvaccinated and in vaccinated children, 1–6 years old in Poland.

Eleven different serotypes were found. Serotypes 23A and 23B constituted 41.5% of the isolates (Figure 1). Serotypes belonging to pneumococcal conjugated vaccines—PCV10 and PCV13—constituted 4.9% and 17.1% of the isolates, respectively. Among pneumococci isolated from vaccinated children, none belonged to PCV10 serotypes and 10.3% of isolates belonged to PCV13 serotypes, whereas pneumococci isolated from the unvaccinated group in 16.7% and 33.3% belonged to PCV10 and PCV13 serotypes, respectively, with insignificant differences when compared to the coverage of PCV10 and PCV13 serotypes between vaccinated and unvaccinated groups (*p* = 0.08 and *p* = 0.16).

### 3.2. Antimicrobial Susceptibility

The pneumococcal isolates were susceptible to all tested antimicrobial agents in 41.5% of children. These strains belonged to serotype 23A (4 isolates), serotypes 10A, 35F/47F (2 isolates), and 6A, 15B, 23B, 35B, (1 isolate per each serotype). Among all of the strains, 34.1% showed decreased susceptibility to penicillin. *S. pneumoniae* isolates were resistant to co-trimoxazole (26.8%), tetracycline (26.8%), erythromycin (31.7%), and clindamycin (14.6%) (Figure 1B). All isolates were susceptible to vancomycin and moxifloxacin. Multidrug resistance was present in 14.6% of the isolates. All MDR-SP were non-susceptible to penicillin. Antibiotic resistant pneumococci were mostly distributed among serotypes not belonging to PCV10 and PCV13 (Figure 1B). PNSSP and MDR-SP strains represented PCV10 serotypes in 4.9% and PCV13 serotypes in 9.8%, respectively. Colonization with PNSSP and MDR-SP strains was found in 13 (31.7%) and 6 (14.6%) children, respectively.

### 3.3. Nasopharyngeal Microbiota by Real-Time PCR

**Figure 2:**
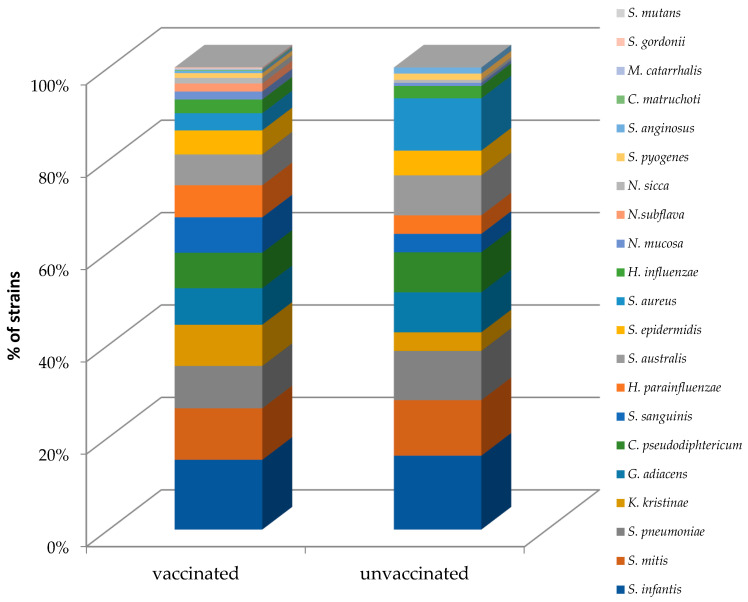
Bacterial frequency analysis in the vaccinated and unvaccinated groups.

In a molecular analysis with real-time PCR, from the 176 patients studied, 643 species/genes of 21 various microbial species were retrieved. In one sample, 0–14 (mean 3.65 ± 2.96) species/genes were detected; 145 (82.4%) children were positive for at least one of the tested microorganisms. The most frequent bacterial species in all groups were *Streptococcus infantis*, *S. mitis*, *S. pneumoniae*, *Granulicatella adiacens*, and *C. pseudodiphtericum*. A difference in the mean numbers of the bacterial species detected in one sample in the vaccinated (3.92 ± 3.054 range 0–14) and non-vaccinated (3.08 ± 2.7, range 0–12) children was observed without statistical significance (*p* = 0.076). Figure 2 presents the bacterial frequency analysis in the vaccinated and unvaccinated groups.

**Figure 3:**
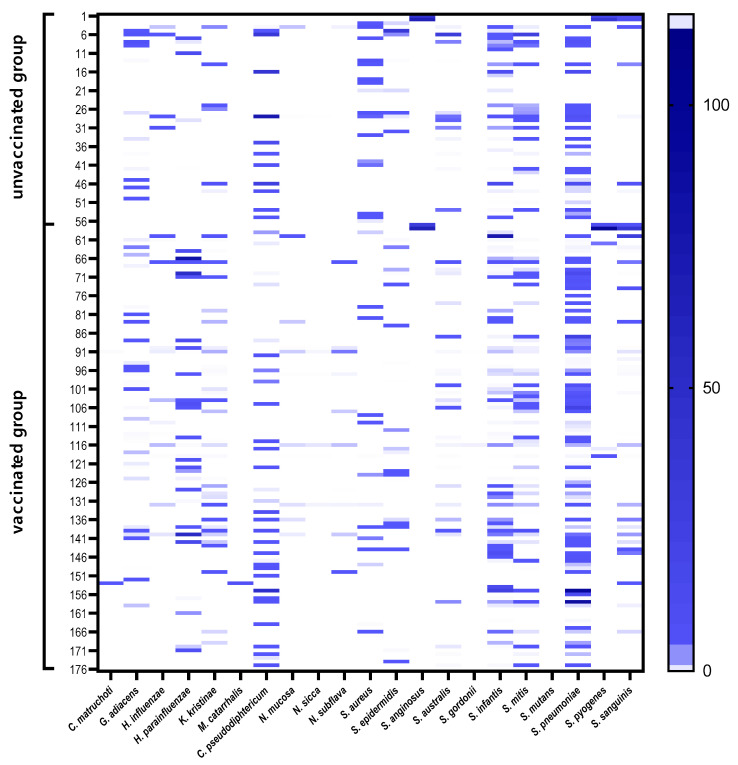
Heat map showing the relative abundance of species/genera genes across 176 samples. For identification of microbial species or microbial genes, the ΔCT method was used. The 2^-^^ΔCT^ data transformation was used.

**Figure 4:**
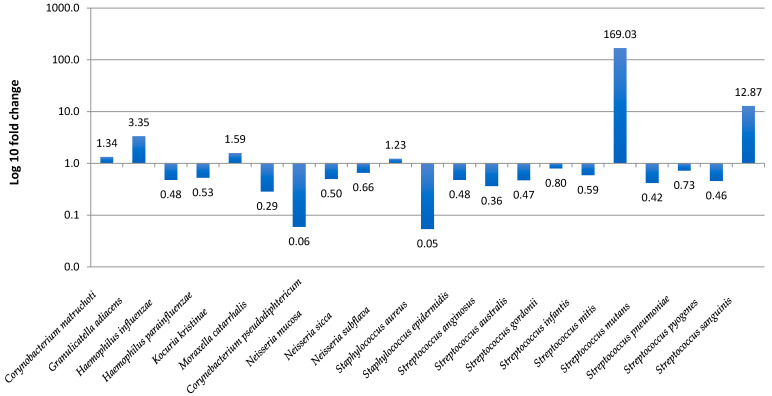
Accurate profiling of pathogenic and commensal microbes in vaccinated and unvaccinated children. Foldchange in microbial species abundance (vaccinated/unvaccinated groups) was calculated by the ∆∆CT method using the Pan Bacteria 1 genomic DNA to normalize. At least a 5- to 10-fold increase or decrease in relative abundance may be considered significant.

The prevalence of URTI pathogens in children, namely *S. pneumoniae*, *H. influenzae*, *M. catarrhalis*, and *S. aureus* was 30.1%, 9.1%, 0.6%, and 18.2%, respectively. The statistical analysis revealed the differences in prevalence of several species in vaccinated and unvaccinated children. *Kocuria kristinae* (*p* = 0.0046), *S. sanguinis* (*p* = 0.028), and *H. parainfluenzae* (*p* = 0.048) were significantly more frequently present in the vaccinated group, whereas *S. aureus* (*p* = 0.0062) was significantly associated with the unvaccinated group. Microbial profiles showing bacterial composition and relative abundance of nasopharyngeal samples are presented in Figure 3. The ΔΔCT method was used for the relative profiling and comparison between two populations from vaccinated and unvaccinated children. Microbial DNA qPCR array correlated with increased amounts of *Streptococcus mitis* and *S. sanguinis* in vaccinated children, with reduced amounts of *C. pseudodiphtericum*, *S. aureus*, and *M. catarrhalis* (Figure 4).

The relative abundance of bacterial species was found to be similar between the vaccinated and unvaccinated groups for the majority of the species. Mann–Whitney tests showed significantly different relative abundance in both groups and were confirmed with positive associations of *K. kristinae* (*p* = 0.0063) and *S. sanguinis* (*p* = 0.016) in the vaccinated group as well as a negative association of *S. aureus* (*p* = 0.0088) in this group.

### 3.4. Prediction of Bacterial Communities Profile

**Figure 5:**
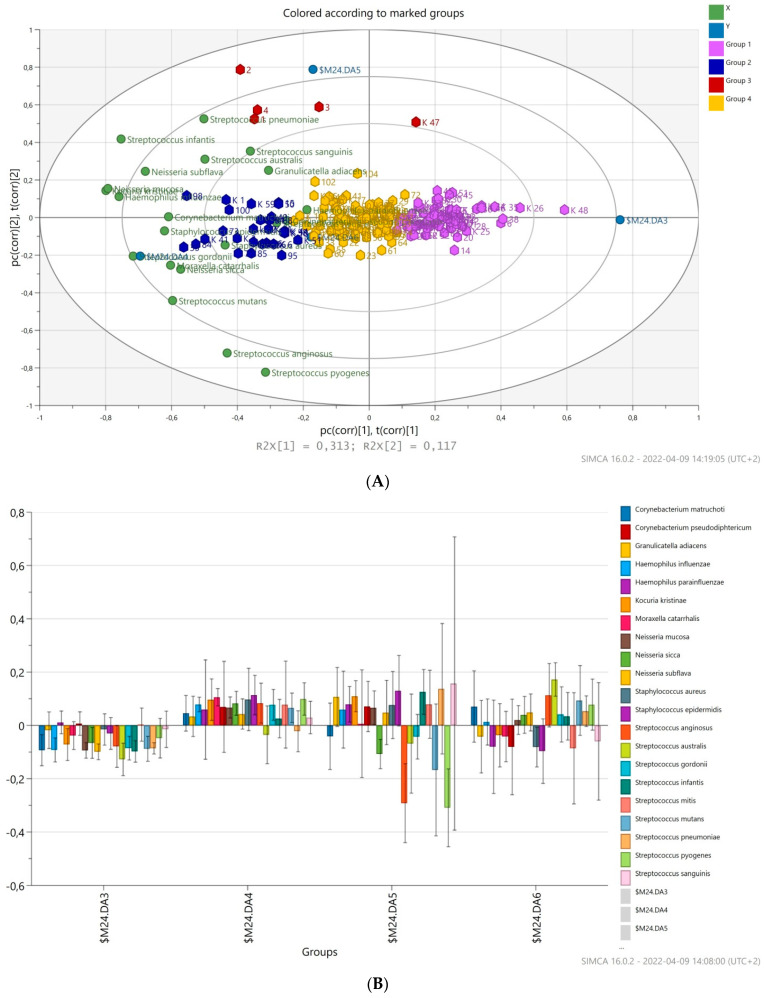
PLS-DA with nasopharyngeal samples was performed based on the 21 bacterial species tested. (**A**) Biplot of PLS-DA with nasopharyngeal samples. Different colors of points in plots were represented for different groups. (**B**) Coefficient overview plot displays how bacterial species take part in four created groups: M17DA3, violet; M17DA4, blue; M17DA5, red; M17DA6, yellow.

The principal component analysis (PCA) was applied to compare the overall structure of nasopharyngeal microbiota of all samples using data scaled to UV (Figure 5). The built model explains 48.9% of the variations. The first principal component explained 31.3% of the overall variability, whereas the second principal component explained 11.0% of the variability. Clustering by vaccination status was not observed for tested nasopharyngeal samples. As shown in Figure 5, the variables placed closer to the circle were more correlated with the component. Most observations are close to the plot origin, showing rather average properties. The first PC was significantly positively correlated with *G. adiacens*, *N. subflava*, *S. infantis*, and *S. pneumoniae*. A significant positive correlation of the second PCs with *S. pyogenes*, *S. mutans*, *S. sanguinis*, and *M. catarrhalis* was observed. PCA identified four distinct clusters of microbiota profiles correlating strongly with a predominance of species, respectively, as well as connected by a group of community profiles representing mixed microbiota.

A hierarchical cluster analysis based on the relative abundance of species revealed a separation of four groups of samples (red group, violet group, blue group, and yellow group, Figure 5) on the basis of the first two principal component (PC) scores. This discrimination was also confirmed by a discriminant analysis (PLS-DA). According to PLS-DA, species, such as *H. influenzae*, *K. kristinae*, *N. mucosa*, and *S. infantis* were markedly high in the red group, with *N. sicca*, *S. anginosus*, and *S. pyogenes* negatively correlated. The blue group of patients had abundance of different microbial species in the nasopharynx; *S. gordonii*, *S. anginosus*, *S. epidermidis*, *N. mucosa*, *N. sicca*, *K. kristinae*, *M. catarrhalis*, and *H. influenzae*. *S. australis* contributed mainly to the yellow group of patients but *S. aureus* was negatively associated. The violet group of patients was markedly colonized by low amounts of bacteria tested, reported by a negative association of most of them.

## 4. Discussion

Our research study is the first of its kind in Poland and one of the few in the world, as it concerns pneumococcal carriage in healthy children in the PCV era. In Poland, data regarding the influence of vaccination on prevalence, antibiotic resistance, and serotype distribution of *S. pneumoniae* isolates are limited [21,22]. We have shown the impact of the introduction of PCVs on nasopharyngeal SP carriage in young children, with serotype distribution after a 3-year period of PCV implementation in a national immunization schedule.

Our data from the pre-vaccination sampling period presented the rate of presence of *S. pneumoniae*, ranging from 33% to 44% (2002–2003) in healthy preschool children [23,24] and from 64.1% to 70.2% (2011–2012) in children with recurrent upper respiratory tract infections [25,26]. In this study, pneumococcal colonization in healthy children aged 1–6 years was at least two times lower (23.3%), but no difference in carriage prevalence in vaccinated and unvaccinated groups was observed due to replacement with non-vaccine-type (NTV) pneumococci. In England, however, the prevalence of pneumococcal carriage in children <5 years studied in 2012 and 2013 after the PCV13 implementation and compared with that in two previous periods, 2001–2002 before the PCV7 introduction and 2008–2009 after the PCV7 introduction, was highly similar—47.7%, 51.0%, and 48.4%, respectively [27]. Since the introduction of PCVs, a global reduction in vaccine-type pneumococci carriage has previously been demonstrated elsewhere among the vaccinated and the unvaccinated populations following PCV vaccination [28,29]. We found a major reduction in the PCV serotype carriage in vaccinated children from 1 to 6 years old. In our study, we also observed a cohort effect, showing reductions in the PCV serotype carriage independent of PCV vaccination, which may indicate herd immunity. Moreover, the non-vaccine serotype carriage rate was high, independent of PCV vaccination (89.7% and 66.7% in vaccinated and unvaccinated groups, respectively). 

In our pre-vaccination carriage studies, 51.0–73.4% and 62.7–80.4% of the isolates belonged to serotypes present in the 10- and 13-valent conjugate vaccine, respectively [23,25]. These percentages of PCV serotypes decreased spectacularly to 4.9% and 17.1% of the isolates, respectively.

The introduction of PCV13 was monitored in several studies in the nasopharyngeal carriage, showing temporal reduction trends in the nasopharyngeal carriage of PCV13 serotypes in PCV13-vaccinated children compared with PCV13-unvaccinated individuals within 1 year of PCV13 introduction [30]. A report from England about observations made for the first three study winters during which only PCV7 had been introduced, through two further winters surrounding PCV13 introduction, showed a stable overall prevalence of pneumococcal carriage (30%). Vaccine serotypes decreased (*p* < 0.0001) with concomitant increases in non-vaccine serotypes. Significant decreases for vaccine serotypes 6B, 19F, 23F (PCV7), and 6A (PCV13), and increases for non-vaccine serotypes 21, 23B, 33F, and 35F, were detected [31]. In a study from Greece, carried out in 2010–2011, non-PCV13 serotypes accounted for 73.1% of the isolates; 23B, 15B/C, 16F, 21, 11A, 15A, 6C, 10A, 22F, and 23A were the most common [32]. The impact on NP carriage and invasive disease of *S. pneumoniae* was estimated after the introduction of the 13-valent PCV in March 2010 in Atlanta, GA, USA [33]. The proportion of pneumococcal carriage accounted for by non-PCV13 serotypes (excluding 6C) increased from 68.4% to 96.9% (*p* < 0.0001). Non-PCV13 serotypes 35B, 15B/C, 11A, 21, 23B, and 15A were the most commonly carried serotypes during the last two study periods. Carriage of serotype 35B significantly increased during the six study periods from 8.9% to 25.3% (*p* < 0.05). In our study, 23B was dominated serotypes followed by 23A, 10A, 6A, and 35B.

Epidemiological studies suggest that such a change in serotype frequencies is often connected with an increase of antibiotic resistance among nonvaccine serotypes. Obolski et al. [34] built multi-locus models for the bacterial pathogen population structure and developed a theoretical framework incorporating variations of serotype and antibiotic resistance to examine how their associations may be affected by vaccination. Using this framework, they found that vaccination could result in a rapid increase in the frequency of preexisting resistant variants of nonvaccine serotypes due to the removal of competition from vaccine serotypes [34]. The results reported antibiotic resistance in *S. pneumoniae* in nine European countries, showing that pneumococcal vaccination was associated with an increase in the prevalence of pneumococcal antimicrobial resistance [35]. This was proof that vaccination might facilitate the appearance of new pneumococcal serotypes, which are more resistant to antimicrobial agents [36] due to the replacement of vaccine serotypes [37]. In a study carried out within 3 years after introduction of PCV-13 in USA Serotype 35B, 6C and nontypeable isolates demonstrated moderate nonsusceptibility to the selected antibiotics. A total of 30% of isolates tested were resistant to erythromycin, followed by 26% resistant to cefuroxime and 12% resistant to clindamycin. The overall penicillin and ceftriaxone nonsusceptibility rates were 10% and 8%, respectively, but 83% and 81% for serotype 19A [33]. In our studies provided in 2002–2003, high penicillin, erythromycin, clindamycin nonsusceptibility was detected (39.2, 29.5%, 29.2%, respectively [24]. Multidrug resistance was common (35.7%); 97.5% of drug-resistant isolates represented serotypes, including the 10- and 13-valent conjugate vaccines [23]. Similar findings were shown in 2011–2013 where PNSSP and MDR-SP strains represented PCV10 serotypes in 83.9% and 80.3%, respectively, and PCV13 serotypes in 89.3% and 88.5%, respectively [38,39]. In this study, PNSSP and MDR-SP strains belonged to non-PCV10 serotypes in 95.1% and non-PCV13 serotypes in 90.2%, respectively. Moreover, in this study, nonsusceptibility to penicillin (34.1%) and erythromycin (31.7%) was not any lower. The high rates of resistance to erythromycin were similar to the other studies [33,40,41]. This has strong clinical implications as many GPs prescribe macrolides for the treatment of upper respiratory infections in children. All isolates were susceptible to vancomycin and levofloxacin in our study population, as in the others [33,40].

The rates of asymptomatic carriage markedly varied between different age groups in our study (*p* = 0.0038). These findings were previously observed by others [42,43]. However, in other studies, the highest rates of asymptomatic colonization and the widest range of serotypes were expressed in groups of young children aged 1.5–3 years [41,42], in our study, this finding was shown in a group of children aged 3–4 years. This could be due to the difference of percentage of vaccinated children in the three tested group, which were 84%, 70.7%, and 60.5% for groups of 1–2 years, 3–4 years, and 5–6 years old children, respectively. In the youngest group, the pneumococcal carriage was the least.

PCVs may alter nasopharyngeal bacterial composition and diversity; however, these findings have not been consistent [44,45,46,47]. A Dutch study reported that the PCV7 was associated with shifts in microbial composition and increases in bacterial diversity in children at 12 months of age but not at 24 months of age [45]. The study in healthy infants over the first year of life in Switzerland determined that children vaccinated in the PCV13 era had higher microbial diversity and microbiota stability than children vaccinated in the PCV7 era and that lower pneumococcal carriage prevalence in the PCV13 era was detected [44]. In Swiss children younger than 2 years old, with acute otitis media, PCV7 reduced the prevalence of commensal families [48]. However, no effect on the microbiome of Kenyan children (aged 12–59 months) 180 days after PCV10 vaccination was observed [47]. In our study, microbiota of vaccinated children was more diverse with dominance of the commensal species.

Some studies have reported an inverse correlation between the nasopharyngeal carriage of *S. pneumoniae* and *S. aureus* [26,49]. Positive associations were reported between pair-wise combinations of *S. pneumoniae* and *H. influenzae* and between *S. pneumoniae* and *M. catarrhalis* but not for *S. pneumoniae* and *S. aureus*, or *H. influenzae* and *S. aureus* [16,26]. A previous study indicated that vaccination against a common colonizer affects microbiota composition and structure [45]. We observed a significant reduction in carriage by *S*. *aureus* among PCV-vaccinated children. A negative association between pneumococci (both PCV-7 serotypes and non-PCV-7 serotypes) and *S. aureus* was also found in a randomized controlled trial on the effect of PCV7 on nasopharyngeal carriage [50]. We found slightly higher rates of pneumococcal carriage among unvaccinated children but the difference was not significant. Nasopharyngeal samples of PCV-vaccinated children detected significantly higher abundances of *Streptococcus* spp. (*S. mitis/sanguinis*). It seems that the vaccination acts strongly at the herd immunity level, which decreases the *S. pneumoniae* pool in the population and pneumococcal carriage in unvaccinated children. Data presented in this study can help to assess the effectiveness of the PCV vaccine and can be used to help guide and establish recommendations for antibiotic therapy for pneumococcal disease in the future. This study also showed that the introduction of PCV13 instead of PCV10 into the national immunization program would decrease the circulation more of the vaccine serotypes. Information about the carriage rate and serotype distribution among Polish children obtained during this study can be used as a baseline in future carriage projects, including these evaluating the influence of mass migration to Poland.

Our study has some limitations. First, we swabbed 176 participants and the power to detect the carriage of serotypes with a low prevalence was limited. The second limitation is that we only recruited participants from one Polish region and, therefore, cannot be certain that they are representative of the whole country. On the other hand, conducting repeated carriage studies in the same regions using a comparable methodology provides longitudinal carriage data for the same population, which should allow a more accurate assessment of vaccine impact over time.

## 5. Conclusions

Introduction of PCV for routine infant immunization was associated with significant reductions in the nasopharyngeal carriage of PCV serotypes and resistant strains amongst vaccine serotypes. However, carriage of non-vaccine pneumococcal serotypes increased modestly, particularly serotype 23B. Moreover, the antibiotic resistance of isolated pneumococcal strains was not much lower, showing PNSSP strains equally reported in vaccinated and unvaccinated children. Further investigation is necessary to determine whether nonvaccine pneumococcal serotypes carried in the nasopharynx are associated with a significant increase of antibiotic resistance and domination in pneumococcal diseases.

## Figures and Tables

**Table 1:** Demographic and clinical characteristics of 176 healthy children.

Parameters		Sp Colonized (n = 41)	Sp Uncolonized (n = 135)	OR (95%CI)	*p* Value
Age (years)	1–2	6 (14.6%)	19 (14.1%)	2.0 (0.7–6.5)	0.22
	3–4	25 (61.0%)	50 (37.0%)	3.3 (1.4–7.5)	0.0038
	5–6	10 (24.4%)	66 (48.9%)	referent	
Sex	Female	16 (39.0%)	63 (46.7%)	0.7 (0.4–1.5)	0.47
	male	25 (61.0%)	72 (53.3%)		
Siblings	No	15 (36.6%)	45 (33.3%)	Referent	
	1	15 (36.6%)	72 (53.3%)	0.6 (0.3–1.4)	0.30
	>2	11 (26.8%)	18 (13.3%)	1.8 (0.7–4.7)	0.22
Passive smoking		3 (7.3%)	19 (14.1%)	0.5 (0.1–1.7)	0.42
Place of residence	Rural	12 (29.3%)	36 (26.7%)	1.1 (0.5–2.5)	0.84
	urban	29 (70.7%)	99 (73.3%)		
DCC/orphanage attendance		34 (82.9%)	118 (87.4%)	0.7 (0.3–1.8)	0.45
Antibiotic therapy	AM/AMC	4 (9.8%)	28 (20.7%)	Referent	
	Macrolides	1 (2.4%)	9 (6.7%)	0.3 (0.07–1.4)	0.14
	Co-trimoxazole	1 (2.4%)	2 (1.5%)	0.2 (0.02–2.5)	0.35
	Cephalosporins	5 (12.2%)	11 (8.1%)	1.1 (0.08–15.2)	1.0
Number of antibiotic therapy	0	28 (68.3%)	85 (63.0%)	Referent	
	1	9 (22.0%)	22 (16.3%)	1.2 (0.5–3.0)	0.65
	>2	3 (7.3%)	26 (19.3%)	0.4 (0.1–1.2)	0.13
RTIs	Pharyngitis	11 (26.8%)	35 (25.9%)	Referent	
	Otitis media	1 (2.4%)	24 (17.8%)	0.3 (0.04–2.6)	0.46
	Sinusitis	0 (0)	13 (9.6%)	0.3 (0.02–5.0)	0.35
	Laryngitis	3 (7.3%)	9 (6.7%)	2.6 (0.6–11.0)	0.19
Number of URTIs	0	12 (29.3%)	36 (26.7%)	Referent	
	1	9 (22.0%)	28 (20.7%)	0.96 (0.4–2.6)	1.0
	>2	19 (46.3)	65 (48.1%)	0.9 (0.4–2.0)	0.83
Hospitalization		10 (24.4%)	25 (18.5%)	1.4 (0.6–3.3)	0.50
Anti-pneumococcal vaccination	total	29 (70.7%)	91 (67.4%)	1.2 (0.5–2.5)	0.85
	PCV 10	10 (24.4%)	37 (27.4%)	0.9 (0.4–2.3)	1.0
	PCV13	15 (36.6%)	51 (37.8%)		
	No data	4 (9.8%)	3 (2.2%)		

Abbreviations: DCC: daycare center; Sp: *Streptococcus pneumoniae*; RTIs: respiratory tract infections; URTIs: upper respiratory tract infections; PCV: pneumococcal conjugate vaccine; OR: odds ratio; CI: confidence interval; AM/AMC: ampicillin/amoxicillin + clavulanic acid. Variables in groups were compared with the Fisher’s exact test.

## Data Availability

Due to privacy and ethical concerns, the data that support the findings of this study are available upon request from the last author (I.K.-G.).
